# An Unusual Suspect: Lymphoepithelial Cyst of the Pancreas

**DOI:** 10.1155/2016/5492824

**Published:** 2016-10-26

**Authors:** Abimbola Adike, Jennifer L. Horsley-Silva, Neha Deval, Christopher R. Conley, Dora M. Lam-Himlin, Cuong C. Nguyen

**Affiliations:** ^1^Mayo Clinic Arizona, Division of Gastroenterology and Hepatology, Scottsdale, AZ, USA; ^2^Mayo Clinic Arizona, Department of Laboratory Medicine and Pathology, Scottsdale, AZ, USA

## Abstract

Lymphoepithelial cysts (LECs) of the pancreas are benign, rare pancreatic cysts that are found predominantly in men. These cysts can present as a diagnostic conundrum given their rarity and difficulty of distinguishing these cysts from those with malignant potential. We present an incidental case of a LEC in a middle-aged man.

## 1. Introduction

Lymphoepithelial cysts are rare, benign cysts that comprise ~0.5% of all pancreatic cysts and occur most commonly in middle-aged men [1, 2]. LECs were first described in 1985 by Lüchtrath and Schriefers in a cyst that appeared histologically similar to a branchiogenic cyst [3, 4]. LECs cysts are considered true cysts as they are lined by squamous epithelium; and a defining feature of this cyst is keratinizing squamous epithelium surrounded by lymphoid tissue. LECs can range in size from 1 to 15 cm, with an average size at presentation of 4.5–6 cm, and can occur in any part of the pancreas [1, 2, 5, 6]. LECs are often very difficult to differentiate from other true pancreatic cysts and may have elevated serum and intracystic tumor markers. Although characteristically benign, these cysts are often surgically treated because of concerning preoperative features.

## 2. Case Description

**Figure 1:**
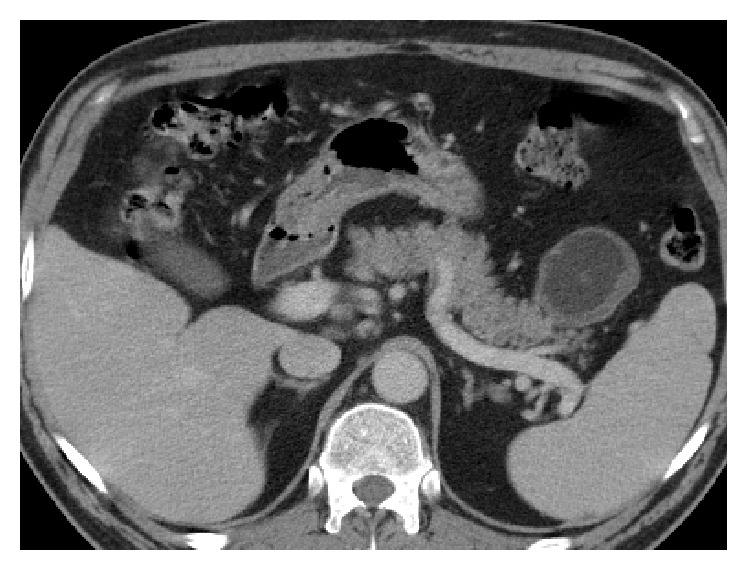
Computed tomography (CT) of the abdomen showing a complex solid cystic mass in the distal pancreatic tail.

**Figure 2:**
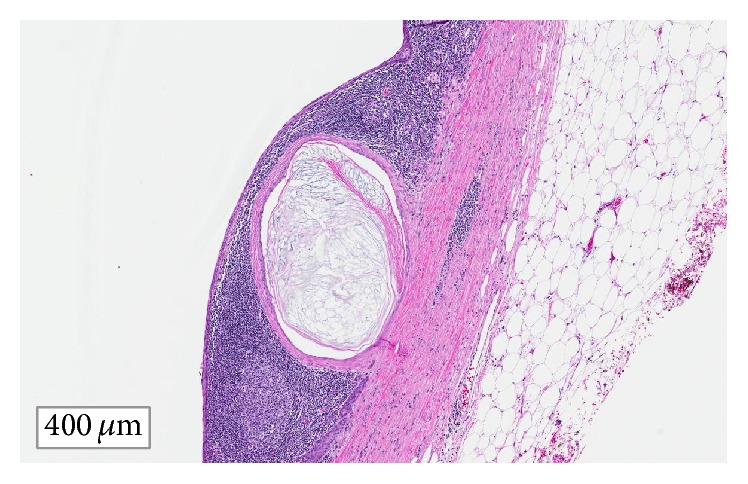
Lymphoepithelial cyst, with squamous cell lining without evidence for mucinous neoplasm or malignancy.

A 56-year-old gentleman presented for evaluation of a pancreatic cyst that had been found incidentally on a CT scan during evaluation for myasthenia gravis (Figure 1). He denied abdominal pain, weight loss, history of diabetes or impaired fasting glucose, and steatorrhea. He was a former smoker and drank 2-3 alcoholic beverages nightly. CT abdomen revealed a complex solid cystic mass in the distal pancreatic tail measuring 7.0 × 9.2 cm without pancreatic or biliary abnormalities and without liver lesions. Endoscopic ultrasound (EUS) demonstrated an anechoic lesion, without ductal communication or dilation, arising from the tail of the pancreas, measuring 8.0 × 8.2 cm in diameter. The cyst had a few compartments, described as thickly septated, and a mural nodule was present. Fine needle aspiration (FNA) showed amorphous debris with squamous epithelial cells, few neutrophils, and lymphocytes. Amylase was 319 U/L and carcinoembryonic antigen (CEA) was 1582 ng/mL. The patient subsequently underwent a distal pancreatectomy and splenectomy with regional lymph node dissection. Pathology demonstrated a benign pancreatic lymphoepithelial cyst measuring 9.3 cm without evidence of malignancy (Figure 2).

## 3. Discussion

LECs of the pancreas are extremely rare types of pancreatic cysts and account for about 0.5% of pancreatic cysts [1]. It occurs predominantly in males, with a male to female ratio of 4 : 1 to 6 : 1, and at a mean age of 55 years [1, 4].

Possible theories of the origin of LECs include formation of the cysts from squamous metaplasia of an intrapancreatic duct that protrudes into a peripancreatic lymph node; derivation from epithelial remnants in lymph nodes; or displacement of branchial cleft cysts and fusion with the pancreas during embryogenesis [1, 2, 4]. It has also been suggested that LECs may be a form of teratoma although the presence and distribution of lymphoid tissue in LECs are unusual for a teratoma [1]. LECs can be found outside of the pancreas and have been described in the parotid, submandibular glands, and thyroid glands. The etiology and pathogenesis of LECs differ by anatomical location [4].

Although the most common presentation of pancreatic LEC is abdominal pain (40% of patients), it is often incidentally found [4, 6]. Patients can also present with malaise, nausea, vomiting, anorexia, and back pain [1]. Weight loss can also be seen, thus, mimicking malignant pancreatic neoplasms [7].

LECs are often round and very well-defined from the pancreas and surrounding adipose tissue on imaging and are often seen protruding from the pancreas parenchyma. Although they have been previously described as predominantly peripancreatic on imaging, Mege et al. found that 79% of LECs were intrapancreatic in a retrospective analysis of 104 patients [1, 6]. LECs can be found in any part of the pancreas but most commonly involve the body and tail of the pancreas, with 40% being unilocular and 60% being multilocular, and they may contain calcifications [1, 2, 6].

The diagnosis of LEC requires accurate differentiation from other cystic neoplasms and is often difficult to differentiate from other cysts such as intraductal papillary mucinous neoplasms and mucinous cystic neoplasms. Preoperative diagnosis may be possible with use of 3D-CT scans, in-and-out of phase MRI scans, and endoscopic ultrasound with needle aspiration of cyst contents. Intracystic tumor markers findings are variable in LECs, with up to 17–32% having an elevated CEA [5, 6, 8]. Patients may also have elevated serum CA 19-9 levels [5, 9]. In a retrospective study of 117 cases of LECs by Mege et al., serum CA 19-9 was elevated in 50% of cases [6]. MRI may be helpful in making a diagnosis of LEC with a high-signal on T1-weighted images based on the lipid component which is diminished on fat-suppressed T1-weighted images [6].

Cystic contents are composed mainly of keratin, with a characteristic “cheesy” or “caseous” appearance [10]. A defining feature of LECs on microscopic evaluation is the presence of squamous epithelium and keratin debris. While treatment can be conservative, surgical resection often occurs to exclude malignancy. In our case, the size of the cyst, elevated CEA, and presence of a mural nodule were initially concerning for malignancy. However, these findings are characteristic for LECs as the average size of LECs is about 4.5–6 cm, they often have elevated intracystic CEA levels, and the presence of a nodule in these cysts can be keratinizing squamous pearls [1, 2, 5, 6].

Unlike other true pancreatic cysts, LECs are benign and have no malignant potential. They can be managed conservatively with excellent outcomes [4, 6]. In the retrospective case series by Mege et al., 13 cases underwent conservative management, leading to no malignant transformation or recurrence that was observed with a median follow-up period of 13 months in patients with available data [6].

This case presentation highlights a very rare pancreatic cyst and the need for accurate diagnosis in order to pursue appropriate treatment.
